# Effect of Honey on Pediatric Radio/Chemotherapy-Induced Oral Mucositis (R/CIOM): A Systematic Review and Meta-Analysis

**DOI:** 10.1155/2022/6906439

**Published:** 2022-03-18

**Authors:** Siyuan Hao, Ling Ji, Yan Wang

**Affiliations:** State Key Laboratory of Oral Diseases & National Clinical Research Center for Oral Diseases &Department of Pediatric Dentistry, West China Hospital of Stomatology, Sichuan University, Chengdu, China

## Abstract

**Background:**

As a common side effect of radio/chemotherapy, oral mucositis severely affects patients' quality of life. Honey has been recommended for adults with radio/chemotherapy-induced oral mucositis (R/CIOM), but its effect for pediatric patients has not been systematically evaluated. Therefore, the aim of this systematic review and meta-analysis was to illuminate whether honey is effective in treating pediatric R/CIOM.

**Methods:**

Two authors searched electronic databases including PubMed, Embase, the Cochrane Library, Web of Science, and Scopus to identify relevant studies, published in English. Then, the outcomes data was extracted from eligible studies and pooled in the meta-analysis.

**Results:**

Totally, five studies containing 316 patients were included in our systematic review and meta-analysis. The result indicated that honey intervention significantly reduced the recovery time (MD = −5.10, 95% CI [−9.60, −0.61], *P* < 0.001, *I*^2^ = 98%, random-effect model) in pediatric patients. Honey also reduced the occurrence of all grades of R/CIOM (RR = 0.19, 95% CI [0.12, 0.30], *P* < 0.001, I^2^ = 0%, fixed-effect model) and the occurrence of grade III and grade IV R/CIOM (RR = 0.18, 95% CI [0.08, 0.41], *P* < 0.001, I^2^ = 7%, fixed-effect model), and the sensitivity analysis showed the results were stable and robust.

**Conclusion:**

Therefore, honey could be a competent candidate for the complementary treatment of pediatric R/CIOM.

## 1. Introduction

Radio/chemotherapy-induced oral mucositis (R/CIOM) is a common inflammatory complication in cancer patients, leading to negative clinical manifestations and reduced quality of life and impacting compliance with anticancer treatment [1]. 34% of patients received conventional radiotherapy and over half received fractionation radiotherapy suffer from severe mucositis (grades III and IV) [2]. Radiotherapy and chemotherapy can also cause severe side effects, leading to mouth ulcers, erythema, pain, and eating disorders, complicated by weight loss and opportunistic pathogen infection [3]. It damages the epithelium of the oral mucosa, destroys the normal barrier structure, and contributes to oral microbiome dysbiosis, which further promotes the occurrence of mucositis [4]. The compliance of patients and their quality of life are seriously affected due to the abovementioned consequences [5]. Nevertheless, existing methods to prevent or treat this disease still fail to achieve satisfactory results [6].

Herbal and traditional treatment has been applied on control of chronic diseases for a long time. For example, Heshmati et al. reported that a biologically active phytochemical ingredient found in turmeric called curcumin was a safe and useful supplement to ameliorate PCOS-associated hyperandrogenemia and hyperglycemia [7]. As a kind of herbal and traditional treatment agent, honey has been reported effective in promoting wound healing, facilitating reepithelization and reducing microbial contamination [8], which may attribute to the organic acids, peptides, phenolics, and enzymes it contains [9].

The Multinational Association of Supportive Care in Cancer (MASCC) and the International Society of Oral Oncology (ISOO) suggested that honey had encouraging potential in adult patients with R/CIOM [10,11]. However, in pediatric patients with R/CIOM, the effect of honey was still controversial. Several studies reported that the application of honey could significantly reduce the recovery time for pediatric R/CIOM patients [12–15]. But, Singh et al. and Mishra et al. indicated that the use of honey could not effectively reduce the occurrence of severe R/CIOM (grade III and grade IV) [15,16]. Therefore, the clinical question we focused in this systematic review and meta-analysis was as follows: whether honey is effective in the treatment of pediatric R/CIOM?.

## 2. Methods and Materials

The protocol of this systematic review and meta-analysis has been registered on PROSPERO (http://www.crd.york.ac.uk/PROSPERO), with an ID: CRD42021247110. We referred to the methods used in our previously published article [17]. The preferred reporting items for systematic reviews and meta-analyses (PRISMA) were as follows [18]. The PRISMA checklist is exhibited in Supplementary File 1.

### 2.1. PICOS

#### 2.1.1. Participants

Participants we included were pediatric patients undergoing chemotherapy or radiotherapy with R/CIOM.

#### 2.1.2. Intervention and Control

The experimental groups received honey agents including the application of honey directly and cubes made of honey. The control group only kept oral hygiene, or received anesthetic agents including benzocaine or lidocaine gel.

#### 2.1.3. Outcomes

The outcomes we focused included recovery time of patients, the occurrence, and the severity of R/CIOM.

#### 2.1.4. Study Type

Only randomized controlled trials were included, regardless of blinding. We only included studies published in English.

### 2.2. Search Strategies

Two authors (Siyuan Hao and Lin Ji) searched a number of databases to collect studies on the relevant issue through following keywords: mucositis, neoplasm, chemotherapy, radiotherapy, pediatric, and honey. Then, we checked the collected studies to identify studies which were potential to be included in our systematic review and meta-analysis. The orientations gave by Salvador-Olivan were followed to avoid errors in search strategies [19]. Search strategy in PubMed is exhibited in Table 1.

### 2.3. Study Selection

All of the search records were downloaded and imported from databases including PubMed, Embase, the Cochrane Library, the Web of Science, and Scopus to EndnoteX9. Firstly, we used the finding duplication function of Endnote software and further removed the duplicate records by manual work. Then, two authors (Siyuan Hao and Ling Ji) independently read the title and abstract of each remaining study to identify studies relevant to the topic of our systematic review and meta-analysis. Thirdly, two authors (Siyuan Hao and Ling Ji) would independently decide which study could be included according to the inclusion and exclusion criteria shown below. Finally, the disagreement on weather a study should be included or not would be resolved by discussing with a third author (Yan Wang).

### 2.4. Inclusion and Exclusion Criteria

Studies meeting the following criteria would be included in our systematic review and meta-analysis:

(1) Randomized controlled trails; (2) the subjects should be pediatrics patients with R/CIOM; (3) the test group consumed honey directly or were applied with cubes made of honey; (4) the control group only kept ordinary oral hygiene or received anesthetic agents including benzocaine or lidocaine gel, without honey. (5) The intervention time was not less than one week. Studies would be excluded on account of any following reasons: (1) the subjects were allergic to honey; (2) the subjects received other kinds of honey products beyond the intervention; (3) the subjects received antibiotic agents during the intervention; (4) the subjects received any other drugs accelerating oral mucosa reparation such as growth factors; (5) duplicates; (6) data on outcome indicators were incomplete and still not available after contacting the authors.

### 2.5. Data Extraction

Siyuan Hao and Lin Ji reviewed the full text of studies and extracted characteristics including the authors' names, publication year, sample size, assessment tool of oral mucositis, details of intervention, and control and type of oncology treatment pediatric cancer patients received. Additionally, the outcome parameters included (1) the recovery time of mucositis; (2) the severity of mucositis; (3) body weight of patients; and (4) isolation result of aerobic bacteria and *Candida*. The mean difference (MD), the risk ratio (RR), and the 95% confidence intervals (CI) of them were extracted. The authors were contacted if we could not obtain the needed information from the published text.

### 2.6. Risk of Bias Assessment

The Cochrane Handbook was used to assess the quality of the included studies. Two investigators independently (Siyuan Hao and Lin Ji) finished the assessment. The risk of bias was assessed for the following domains included: random sequence generation, allocation concealment, blinding of participants and personnel, blinding of outcome assessments, incomplete outcome data, selective reporting, and other bias. For each domain, the risk of bias for each study was assessed according to three categories: low risk, high risk, or unclear risk. Any unresolved divergence was discussed with a third investigator (Yan Wang).

### 2.7. Grading the Quality of Evidence

We then assessed the quality of the evidence for every outcome with the Grading of Recommendations, Assessment, Development, and Evaluations (GRADE) approach [20]. The quality assessments were performed by two independent authors (Siyuan Hao and Lin Ji), and any disagreements were resolved by discussion with a third author (Yan Wang).

### 2.8. Statistical Analysis

The effect of honey on pediatric R/CIOM was evaluated by the pooled MD and RR and their 95% CI. In the meta-analysis, Cochrane's Q statistics and the Higgins I-squared statistic (I^2^) were used to detect heterogeneity [21]. *P* < 0.1  or I^2^> 50% was regarded as statistically significant heterogeneity, and we applied a random-effect model. A fixed-effect model was preferred when *P* > 0.05 or I^2^ < 50%. All these analyses were performed with RevMan 5.4 software.

### 2.9. Trial Sequential Analysis

Trial sequential analysis (TSA) 0.9.5.10 (https://www.ctu.dk/tsa/) was performed to calculate the required information size (RIS), alpha spending function, trial sequential monitoring boundaries for benefits and harms, and futility boundaries assessment. The type I and type II errors were set at 0.05 and 0.2, respectively. The sample size was calculated with statistical power at 80%.

## 3. Results

### 3.1. Search Results

**Figure 1:**
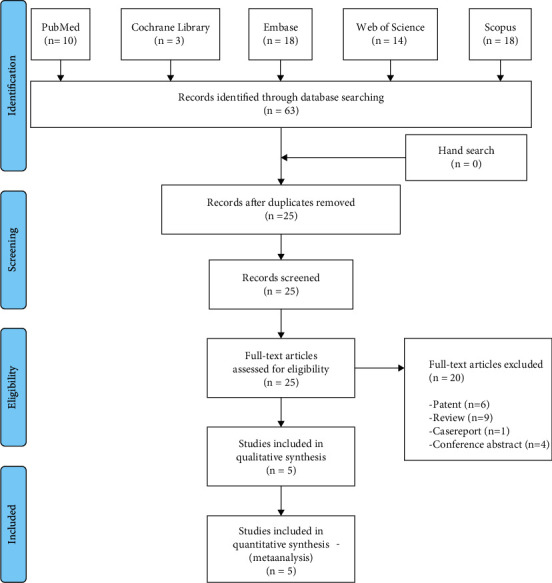
The flowchart of the study selection process.

We identified 10 studies from PubMed, three from the Cochrane Library, 18 from Embase, 14 from the Web of Science, and 18 from Scopus. We had a total of 25 articles after removing duplications. Then, we read the full text of the remaining articles, among which six were excluded because they were patents, nine were reviews, one was a case report, and four were conference abstracts. Ultimately, five studies were selected for systematic review and were included in the meta-analysis (Figure 1).

### 3.2. Characteristics of the Included Studies

The characteristics of the included studies are shown in Table 2. All the studies reported the mean age or age range of the participants, ranging from five to 19 years. The sample size ranged from 40 to 100 patients. The intervention duration varied between five days and 15 days. In four studies, honey was topically applied to the oral mucosa, and one study used ice cube made of honey. The WHO mucositis assessment scale was used in four studies, and Abdulrhman et al. used the National Cancer Institute Common Toxicity Criteria [22].Outcome measures included (1) the recovery time of mucositis; (2) the severity of mucositis; (3) body weight of patients; and (4) isolation result of aerobic bacteria and *Candida*.

### 3.3. Risk of Bias

**Figure 2:**
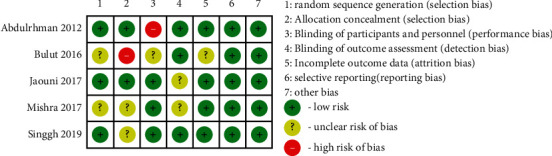
The risk of bias of the included studies was assessed according to the Cochrane Handbook.

Figure 2 shows the assessment of the risk of bias of the included studies. Among the five included studies, two were reported in blinded designs [15, 16]. Two studies claimed that they were blinded trials, but without describing details [13, 14]. Abdulrhman et al. did not blind the subjects (12). All five included studies claimed randomized, three studies were randomized properly [12, 13, 15], and the remaining two studies did not provide a description with available details [14, 16].

### 3.4. The Recovery Duration of R/CIOM

**Figure 3:**
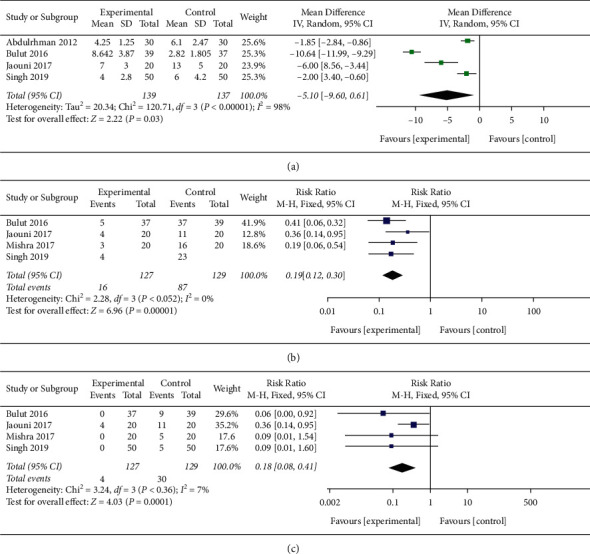
The forest plot of meta-analysis. (a) The recovery time of the honey group was significantly less than that of the control group. (b) The occurrence of R/CIOM in the honey group was significantly less than that in the control group. (c) The occurrence of grade III and grade IV R/CIOM in the honey group was significantly less than that in the control group.

We extracted and pooled the recovery time of R/CIOM from four studies containing 276 subjects. The meta-analysis illuminated that the recovery time of the honey group was significantly shorter than that of the control group (Figure 3(a)) (MD = -5.10, 95% CI [-9.60, -0.61], *P* < 0.001, I^2^ = 98%, random-effect model).

### 3.5. The Occurrence of All Grades of R/CIOM

The occurrence of R/CIOM between the honey group and the control group was compared after the intervention, with four included studies containing 256 subjects [13–16]. The pooled RR indicated that the occurrence of all grades of R/CIOM in the honey group was significantly less than that in the control group (Figure 3(b)) (RR = 0.19, 95% CI [0.12, 0.30], *P* < 0.001, I^2^ = 0%, fixed-effect model).

### 3.6. The Occurrence of Grades III and IV R/CIOM

The impact of honey on the occurrence of grades III and IV R/CIOM was evaluated in four studies containing 256 subjects [13–16]. The results of the meta-analysis of fixed-effect model showed that the occurrence of grades III and grade IV R/CIOM in the honey group was significantly less than that in the control group (Figure 3(c)) (RR = 0.18, 95% CI [0.08, 0.41], *P* < 0.001, I^2^ = 7%, fixed-effect model).

### 3.7. Other Secondary Outcomes

Herein, we qualitatively described the data not suitable to pool in the meta-analysis. Jaouni et al. reported the weight change of patients after the study intervention. Compared with the control group, the honey group had a higher body weight. They also detected aerobic bacteria and *Candida* in oral patients. The counts of aerobic infectious bacteria and *Candida* in the intervention group were also less than those in the control group [13].

### 3.8. Sensitivity Analysis

To assess the stability of the results of our meta-analysis, we did the sensitivity analysis by omitting the included studies one by one. When omitting the included studies one by one, the result of meta-analysis did not dramatically change, showing that our results were stable and robust (Supplementary File 2).

### 3.9. Grading the Quality of Evidence

According to the GRADE approach, we assessed the quality of the evidence for every outcome of our systematic review and meta-analysis. The outcome recovery time was evaluated as low evidence and the occurrence of all grades of R/CIOM and the occurrence of grades III and IV R/CIOM were evaluated as moderate evidence. The detailed GRADE assessment result is shown in Table 3.

### 3.10. Trial Sequential Analysis

TSA for the recovery duration of R/CIOM showed that, although the amount of sample information in the current cumulative study had not reached the expected amount of information, no further studies were needed for verification. TSA for the occurrence of all grades of R/CIOM indicated that the sample size of included studies had reached the expected amount of information and no further studies were needed for verification. However, due to the included studies provided too little information, the alpha spending boundaries could not be calculated by the software. So, the TSA for the occurrence of grades III and IV R/CIOM was not preformed. The graphs of TSA are shown in Supplementary File 3.

## 4. Discussion

Pediatric patients are more likely to develop RIOM/CIOM than adult patients [23]. The incidence of oral mucositis in children is more than half, or even up to 81%, regardless of the type of antitumor therapy received [24].

To identify the effect of honey on pediatric R/CIOM, we performed this systematic review and meta-analysis that included five RCTs with 316 subjects. The combined data from four studies indicated that honey could effectively reduce the recovery time of pediatric R/CIOM, with low evidence evaluated by GRADE. On the issue of reducing the severity of mucositis, honey was proven to significantly reduce the occurrence of all grades of R/CIOM and the occurrence of grade III and grade IV R/CIOM in pediatric patients compared with the control group, with moderate evidence evaluated by GRADE. Jaouni et al. reported that compared with the control group, the honey intervention group had a higher body weight. It might also contribute to preventing opportunistic infection by aerobic bacteria and *Candida*, improving the ecological balance of the oral microenvironment. These encouraging results suggested that honey could be a competent candidate for the complementary treatment of pediatric R/CIOM.

We noticed that Friend et al. performed a literature review in 2016 and concluded that Grade C evidence supported that honey is effective as a preventative and therapeutic measure for OM in pediatric oncology patients [25]. However, on the one hand, they included both RCTs and observational studies, which made their grade of evidence considerably low. On the other hand, they did not carry out quantitative analysis, which made their conclusion limited. Additionally, our study included RCTs published in recent years and performed a systematic review and quantitative meta-analysis to comprehensively identify the effect of honey on pediatric R/CIOM from evidence with higher quality.

Honey has been used as a kind of therapeutic agent since ancient times. According to previous reports, honey has a wide variety of compounds, including phenols, peptides, organic acids, enzymes, and maillard reaction products, so honey has an inhibitory effect on over 60 kinds of bacteria and fungi. Its antioxidant capacity is important in disease conditions [26,27]. In detail, Massaro [28] reported that the anti-inflammatory mechanism in stingless bee products inhibits the 5-LOX enzyme, which is responsible for the synthesis of proinflammatory mediators. Currently, experimental studies have suggested that honey has the potential to maintain the integrity of epithelial tissue, preventing intercellular rupture [29]. It is worth noting that honey has been reported to inhibit bacterial growth due to its high viscosity, acidic pH, and hydrogen peroxide [30]. Our study indicated that honey is effective in the treatment of pediatric R/CIOM. As a complementary treatment agent, it is also important for pediatric R/CIOM patients to maintain good oral hygiene habits while applying honey.

There also exist several limitations in our systematic review and meta-analysis: (1) there exists significant heterogeneity among the included studies perhaps due to various regimens, doses, duration, center settings, populations enrolled, and so on. (2) The quality of the included studies was relatively poor and can be significant sources of bias. (3) Each meta-analysis contained a small number of studies. The evidence to support it is low and should be noticed. Therefore, the results of our systematic review and meta-analysis should also be interpreted cautiously. Further high-quality, multicenter clinical trials are still required.

## 5. Conclusion

Available evidence demonstrates that honey can significantly reduce the recovery time, the occurrence of all grades of R/CIOM, and the occurrence of grade III and grade IV R/CIOM in pediatric patients. Therefore, honey could be a competent candidate for the complementary treatment of pediatric R/CIOM.

## Figures and Tables

**Table 1:** The search strategy used in PubMed.

Number	Search terms
#1	Stomatitis [MeSH terms]
#2	Stomatitis [Title/Abstract] OR Oral Mucositis [Title/Abstract] OR Stomatitides[Title/Abstract] OR Mucositides, Oral [Title/Abstract] OR Oral Mucositides [Title/Abstract] OR Oromucositis [Title/Abstract] OR Oromucositides [Title/Abstract] OR Mucositis, Oral [Title/Abstract] OR stomati^*∗*^ [Title/Abstract] OR mucositi^*∗*^ [Title/Abstract] OR oromucositi^*∗*^ [Title/Abstract] OR Mouth Mucosa [Title/Abstract] OR Mucosa, Mouth [Title/Abstract] OR Oral Mucosa [Title/Abstract] OR Mucosa, Oral [Title/Abstract] OR Buccal Mucosa [Title/Abstract]
#3	Neoplasms [MeSH Terms]
#4	Neoplasms [Title/Abstract] OR cancer[Title/Abstract] OR neoplasm [Title/Abstract] OR leukemia [Title/Abstract] OR lymphoma [Title/Abstract] OR head and neck cancer [Title/Abstract]
#5	Drug Therapy [MeSH Terms]
#6	Chemotherapy [Title/Abstract] OR Chemotherapies [Title/Abstract] OR Pharmacotherapy [Title/Abstract]
#7	Radiotherapy [MeSH Terms]
#8	Radiotherapies [Title/Abstract] OR Radiation Therapy [Title/Abstract] OR Radiation Therapies [Title/Abstract]
#9	Honey[MeSH Terms]
#10	Honey [Title/Abstract] OR Honeys [Title/Abstract]
#11	Pediatrics[MeSH Terms]
#12	Pediatric [Title/Abstract] OR Pediatrics [Title/Abstract] OR child [Title/Abstract] OR children [Title/Abstract]
#13	1# OR #2
#14	(#3 OR #4) OR (#5 OR #6) OR (#7 OR #8)
#15	#9 OR #10
#16	#11 OR #12
#17	#13 AND #14 AND #15 AND #16

**Table 2:** The characteristics of included studies.

Study	Age	Sample size (T; C)	Type of oncology treatment	Oral mucositis assessment tool	Intervention	Control	Outcomes
Abdulrhman, 2012	Mean 6.9	60 (30; 30)	Methotrexate, 2 g/m^2^, every two weeks	National Cancer Institute Common Toxicity Criteria	*Trifolium alexandrinum* honey, 0.5 g/kg, 3 times daily	Benzocaine 7.5% gel, 3 times daily	Recovery time
Bulut, 2016	6–17	76 (39; 37)	Methotrexate treatment	WHO Mucositis Assessment Scale	Wildflower honey, 1 g/kg daily	Routine mouth care	Recovery time, mucositis severity
Jaouni, 2017	Mean 8	40 (20; 20)	Radio/chemotherapy	WHO Mucositis Assessment Scale	Local commercial Saudi honey, 3 times daily	Lidocaine gel	Recovery time, mucositis severity, body weight, aerobic, bacterial, and *Candida*
Mishra, 2017	5–19	40 (20; 20)	5-F uracil or methotrexate treatment	WHO Mucositis Assessment Scale	Ice cubes made of honey, 5 minutes before chemotherapy	Plain ice cubes	Mucositis severity
Singh, 2019	Mean 8.7	100 (50; 50)	Chemotherapy	WHO Mucositis Assessment Scale	Commercially available marketed honey, 1–2 ml,4 times daily	Benzalkonium and lignocaine gel	Recovery time, mucositis severity

**Table 3:** The evidence quality of outcomes assessed according to the GRADE approach.

Quality assessment							No. of patients		Effect		Quality	Importance
No, of studies	Design	Risk of bias	Inconsistency	Indirectness	Imprecision	Other considerations	Recovery time	Control	Outcome	95% CI		
*The recovery time*												
4	Randomized trials	Serious	Serious	No serious indirectness	No serious imprecision	None	139	137	MD −5.1	−9.6–0.61	Low	Important

*The occurrence of R/CIOM*												
4	Randomized trials	No serious risk of bias	No serious inconsistency	No serious indirectness	Serious	None	127	129	RR 0.19	0.12–0.30	Moderate	Important

*The occurrence of grade III and grade IV R/CIOM*												
4	Randomized trials	No serious risk of bias	No serious inconsistency	No serious indirectness	Serious	None	127	129	RR 0.18	0.18–0.41	Moderate	Important

## Data Availability

The data supporting this systematic review and meta-analysis are from previously published studies, which have been cited. The processed data are available from the corresponding author upon request.

## Supplementary Materials

Supplementary Materials
*Supplementary File 1.* Table of PRISMA checklist. *Supplementary File 2.* The table of sensitivity analysis. When omitting the included studies one by one, the result of meta-analysis did not dramatically change, showing that our result was stable and robust. *Supplementary File 3.* The graphs of TSA for the three outcomes of our systematic review and meta-analysis: (1) TSA for the recovery duration of R/CIOM; (2) TSA for the occurrence of all grades of R/CIOM; (3) TSA for the occurrence of grades III and IV R/CIOM.
