# Supplementary material for: Effect of Honey on Pediatric Radio/Chemotherapy-Induced Oral Mucositis (R/CIOM): A Systematic Review and Meta-Analysis
Source: Evid Based Complement Alternat Med. 2022 Mar 18;2022:6906439. doi: 10.1155/2022/6906439 (PMC8956378; doi:10.1155/2022/6906439)
Supplement: Supplementary Materials — Supplementary File 1. Table of PRISMA checklist. Supplementary File 2. The table of sensitivity analysis. When omitting the included studies one by one, the result of meta-analysis did not dramatically change, showing that our result was stable and robust. Supplementary File 3. The graphs of TSA for the three outcomes of our systematic review and meta-analysis: (1) TSA for the recovery duration of R/CIOM; (2) TSA for the occurrence of all grades of R/CIOM; (3) TSA for the occurrence of grades III and IV R/CIOM. [file 6906439.f1.zip › 6906439.f1/Supplementary file 2.docx]

Supplementary file 2. The table of sensitivity analysis. When omitting the included studies one by one, the result of meta-analysis did not dramatically change, showing that our result was stable and robust.

| **The recovery duration of R/CIOM** | | | | |
| --- | --- | --- | --- | --- |
| Omitted study | MD | 95%CI | P value | I^2^(%) |
| Abdulrhman 2012 | -6.22 | -12.13--0.31 | 0.04 | 97 |
| Bulut 2016 | -2.92 | -4.79--1.04 | 0.002 | 78 |
| Jaouni 2017 | -1.62 | -3.15--1.10 | <0.001 | 96 |
| Singh 2019 | -5.03 | -5.79--4.27 | <0.001 | 98 |
| **The occurrence of all grades of R/CIOM** | | | | |
| Omitted study | RR | 95%CI | P value | I^2^(%) |
| Bulut 2016 | 0.22 | 0.12-0.39 | <0.001 | 0 |
| Jaouni 2017 | 0.16 | 0.09-0.28 | <0.001 | 0 |
| Mishra 2017 | 0.19 | 0.11-0.32 | <0.001 | 12 |
| Singh 2019 | 0.19 | 0.11-0.33 | <0.001 | 9 |
| **The occurrence of grade III and IV R/CIOM** | | | | |
| Omitted study | RR | 95%CI | P value | I^2^(%) |
| Bulut 2016 | 0.23 | 0.09-0.55 | 0.001 | 0 |
| Jaouni 2017 | 0.07 | 0.01-0.38 | 0.002 | 0 |
| Mishra 2017 | 0.19 | 0.08-0.47 | <0.001 | 25 |
| Singh 2019 | 0.19 | 0.08-0.47 | <0.001 | 25 |
