# Supplementary material for: Effect of Honey on Pediatric Radio/Chemotherapy-Induced Oral Mucositis (R/CIOM): A Systematic Review and Meta-Analysis
Source: Evid Based Complement Alternat Med. 2022 Mar 18;2022:6906439. doi: 10.1155/2022/6906439 (PMC8956378; doi:10.1155/2022/6906439)

Supplementary file 3. The graphs of TSA for the three outcomes of our systematic review and meta-analysis

1. TSA for the recovery duration of R/CIOM


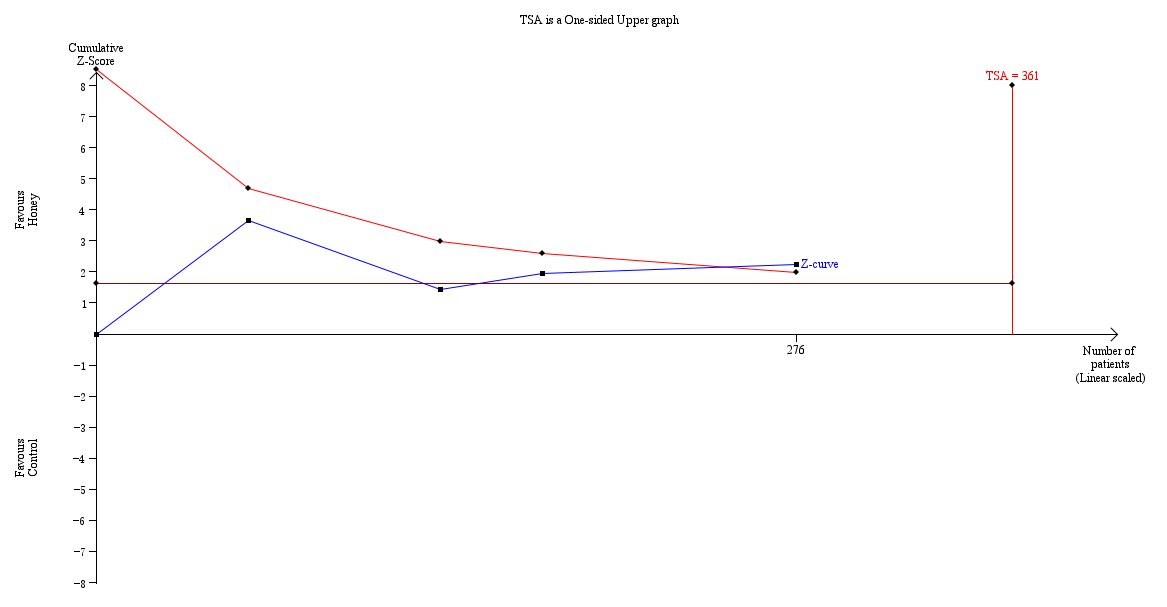


1. TSA for the occurrence of all grades of R/CIOM


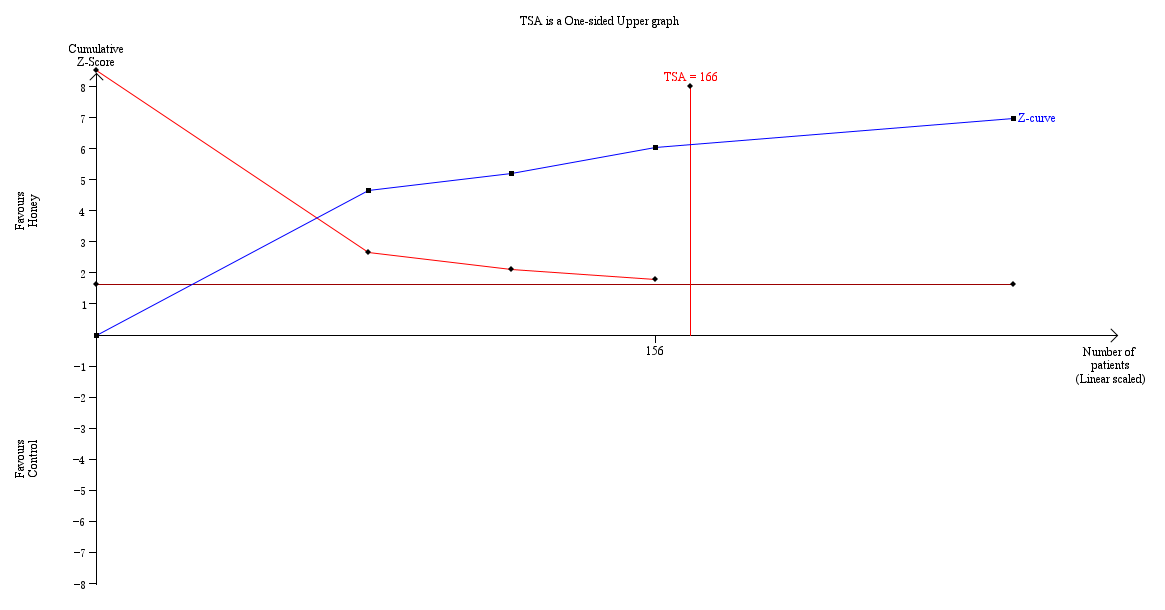


1. TSA for the occurrence of grade III and IV R/CIOM


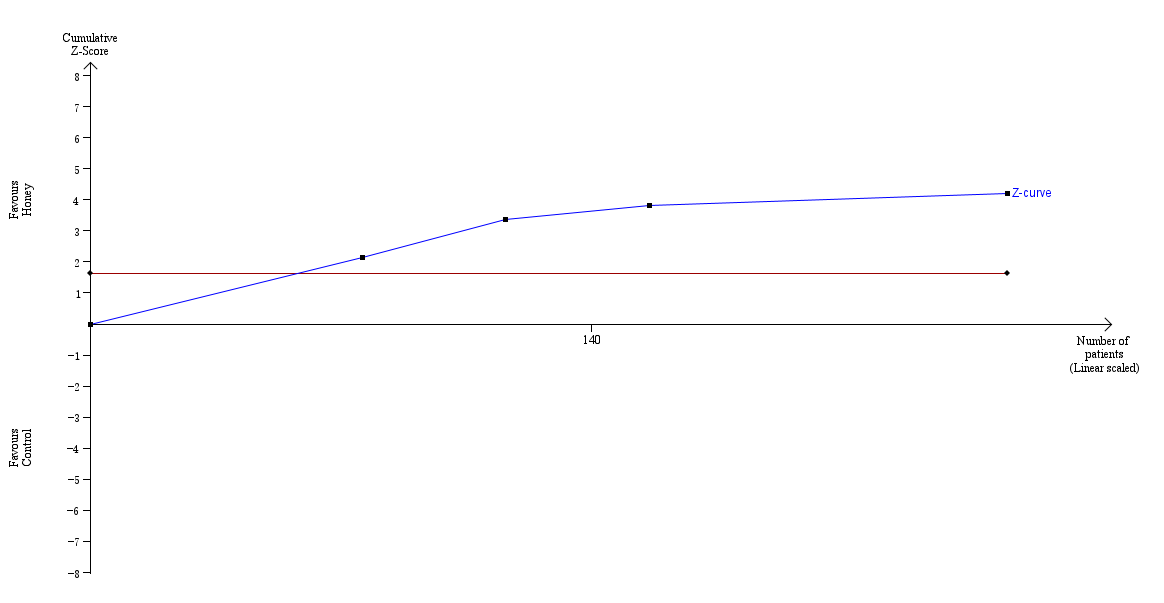

Supplement: Supplementary Materials — Supplementary File 1. Table of PRISMA checklist. Supplementary File 2. The table of sensitivity analysis. When omitting the included studies one by one, the result of meta-analysis did not dramatically change, showing that our result was stable and robust. Supplementary File 3. The graphs of TSA for the three outcomes of our systematic review and meta-analysis: (1) TSA for the recovery duration of R/CIOM; (2) TSA for the occurrence of all grades of R/CIOM; (3) TSA for the occurrence of grades III and IV R/CIOM. [file 6906439.f1.zip › 6906439.f1/Supplementary file 3.docx]
